# Hippocampal transcriptome profiling in a 22q11.2 deletion syndrome mouse model: comparison with human schizophrenia

**DOI:** 10.1186/s13041-026-01300-7

**Published:** 2026-04-05

**Authors:** Hinano Yonemaru, Takaaki Ozawa, Takatoshi Hikida

**Affiliations:** 1 Laboratory for Advanced Brain Functions, Institute for Protein Research, The University of Osaka, Suita, Osaka Japan; 2 Department of Frontier Biosciences, Graduate School of Frontier Biosciences, The University of Osaka, Suita, Osaka Japan

**Keywords:** 22q11.2 deletion syndrome, Schizophrenia, Hippocampus, Transcriptomics, Excitatory-inhibitory balance, Cross-species comparison

## Abstract

22q11.2 deletion syndrome (22q11.2DS) confers one of the highest genetic risks for schizophrenia, yet the molecular mechanisms remain incompletely understood. We performed comprehensive RNA sequencing of the dorsal hippocampus in Df1/+ mice, a 22q11.2DS model, integrating behavioral assessment and cross-species comparison with human schizophrenia postmortem data. Df1/+ mice exhibited selective contextual fear memory impairment without gross locomotor deficits. Transcriptomic analysis using integrated over-representation and gene set enrichment approaches revealed upregulation of synaptic signaling pathways, including glutamatergic and GABAergic neurotransmission, alongside downregulation of translational machinery and ribosomal proteins. Top upregulated pathways included “regulation of postsynaptic membrane potential” and “postsynapse organization,” featuring glutamatergic receptors, voltage-gated channels, and synaptic adhesion molecules. Downregulated pathways centered on protein synthesis, including cytoplasmic translation and ribosome biogenesis. Cross-species comparison with human schizophrenia hippocampus revealed limited but directionally consistent gene-level overlap, with 21 of 23 shared differentially expressed genes showing concordant regulation. “Regulation of postsynaptic membrane potential” was the pathway significantly enriched across both species and analytical methods, encompassing both excitatory and inhibitory receptor subunits and synaptic regulators. Concordantly downregulated genes spanned glial markers and extracellular matrix components. These findings reveal a molecular signature of enhanced synaptic gene expression coupled with reduced translational capacity and glial support, with cross-species correspondence supporting the model’s translational relevance and highlighting excitatory-inhibitory imbalance as a shared mechanism in hippocampal dysfunction underlying schizophrenia.

**Supplementary Information:**

The online version contains supplementary material available at 10.1186/s13041-026-01300-7.

## Introduction

22q11.2 deletion syndrome (22q11.2DS) is the most common microdeletion disorder in humans, occurring in approximately 1 in 4,000 live births [1]. The syndrome arises from hemizygous deletions of a ~ 3 Mb region on chromosome 22q11.2 and manifests with highly variable clinical phenotypes including congenital heart defects, immune dysfunction, craniofacial anomalies, and cognitive impairments [2]. Notably, 22q11.2DS confers the highest known genetic risk for schizophrenia, with approximately 25–30% of individuals developing psychotic disorders in adolescence or early adulthood [3]. This represents a 20- to 25-fold increased risk compared to the general population, making 22q11.2DS a critical genetic model for understanding the neurodevelopmental origins of schizophrenia. Despite this strong genotype-phenotype association, the comprehensive molecular mechanisms linking 22q11.2 deletion to cognitive dysfunction and psychosis risk remain incompletely understood.

Cognitive impairments are prominent features in both 22q11.2DS and schizophrenia, particularly affecting memory, attention, and executive function [4, 5]. Given the central role of the hippocampus in declarative memory formation and spatial navigation, hippocampal dysfunction is considered as a core contributor to these deficits and is consistently implicated in schizophrenia [6]. Neuroimaging studies have revealed reduced hippocampal volume and altered functional connectivity in individuals with 22q11.2DS, correlating with cognitive performance and psychiatric symptoms [7–10].

Mouse models with orthologous deletions, such as Df1/+ and Df(16)A+/- mice, exhibit hippocampus-dependent memory deficits, providing tractable systems for mechanistic investigation of 22q11.2DS-related cognitive dysfunction [11–17]. While several 22q11.2 genes have been individually characterized [18–20], systems-level transcriptomic signatures and their correspondence with human schizophrenia remain largely unexplored. Understanding the transcriptional landscape of the hippocampus in these models may illuminate pathogenic mechanisms and identify potential therapeutic targets. Prior transcriptomic analyses in these models, however, have either been limited to microarray or targeted qPCR approaches, lacking genome-wide resolution and network level inference [16, 21, 22]. Moreover, the extent to which molecular alterations in mouse models recapitulate changes observed in human schizophrenia hippocampus has not been thoroughly evaluated. Cross-species comparison of transcriptional signatures is essential for validating animal models and identifying conserved disease mechanisms that may translate to human pathophysiology. Recent advances in RNA sequencing (RNA-seq) technology and the availability of human postmortem brain transcriptomic datasets now enable such comparative analyses.

In this study, we performed comprehensive transcriptomic profiling of the dorsal hippocampus in Df1/+ mice [12], coupled with behavioral assessment of hippocampus-dependent memory and cross-species comparison to human schizophrenia. We employed a multi-layered analytical approach integrating over-representation analysis (ORA) and gene set enrichment analysis (GSEA) to identify molecular alteration induced by 22q11.2 deletion. To evaluate translational relevance, we compared our findings with postmortem hippocampal transcriptomes from individuals with schizophrenia [23], enabling identification of shared molecular signatures across species. This approach provides molecular insights into hippocampal dysfunction in 22q11.2DS and highlights potential mechanisms underlying cognitive impairments in schizophrenia.

## Results

### Df1/+ mice exhibit selective hippocampus-dependent memory deficits

**Fig. 1:**
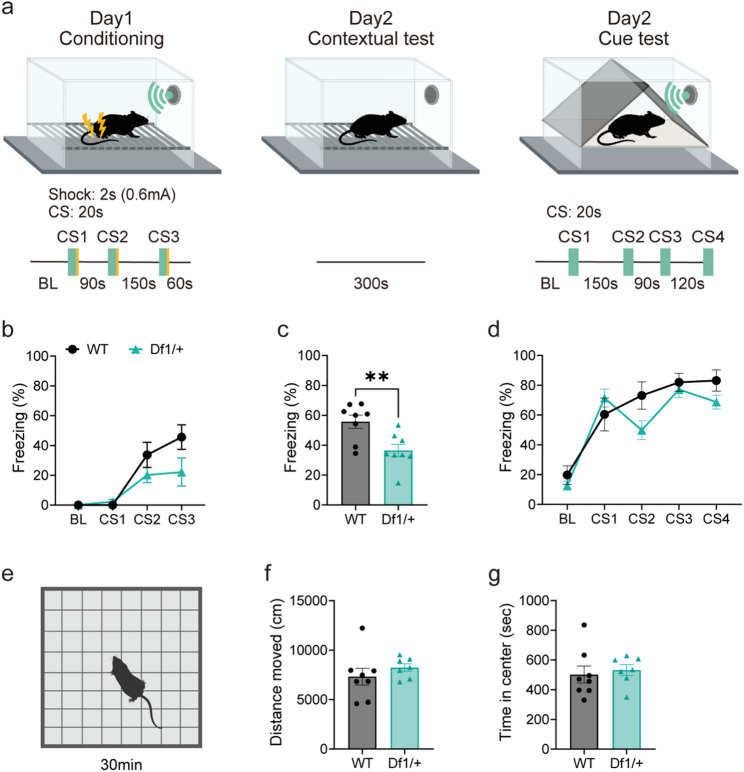
Df1/+ mice exhibit selective hippocampus-dependent memory deficits. **a**–**d** The result of fear conditioning test. Schematic illustration of the fear conditioning (**a**). The percentage of freezing time on the conditioning (**b**), the contextual test (**c**), and the CS test (**d**). WT *n* = 8, Df1/+ *n* = 8. **e–g** The result of open field test. Schematic illustration of the open field test (**e**), the total distance moved (**f**), and the time in center zone (**g**) during 30 min test time. WT *n* = 8, Df1/+ *n* = 7. Data are expressed as mean ± SEM. Two-way repeated measures ANOVA or unpaired *t* test was performed. Asterisks indicate significance: **p < 0.01. Schematic illustrations were created with BioRender.com. Raw data and full statistical results are provided in Additional file 2 and Table S2-1

To assess hippocampus-dependent associative memory, we employed a contextual fear conditioning paradigm comprising conditioning, contextual, and cued sessions (Fig. 1a). During the conditioning phase on Day 1, both wild-type (WT) and Df1/+ mice exhibited a progressive increase in freezing across successive conditioned stimulus (CS) presentations, indicating successful acquisition of the conditioned fear response (Fig. 1b, S1a; main effect of cue, *p* < 0.0001). Raw data and full statistical results are provided in Additional file 2 and Table S2-1.

On Day 2, contextual fear memory was significantly impaired in Df1/+ mice compared to WT littermates, as evidenced by a marked reduction in freezing behavior during the contextual recall test (Fig. 1c, S1b; *p* = 0.0066). In contrast, no significant genotype difference was observed during the cued test (Fig. 1d, S1c), suggesting that the associative memory deficit in Df1/+ mice was specific to the contextual component of fear conditioning rather than general fear reactivity to the auditory cue. These results indicate selective impairment of hippocampus-dependent memory retention in Df1/+ mice, consistent with previous reports [14].

In the open field test, no significant differences were observed between WT and Df1/+ mice in total locomotor activity, indicating that the observed behavioral changes in the fear conditioning task were not attributable to alterations in general motor function or anxiety-like behavior (Fig. 1e-g, S1d, e). In addition, we confirmed the enhanced locomotor response to the psychostimulant MK-801 in Df1/+ mice (Fig. S1f-h; main effect of genotype, *p* = 0.0268), consistent with previous reports [19, 24].

### Transcriptomic profiling reveals distinct gene expression patterns in Df1/+ hippocampus

**Fig. 2:**
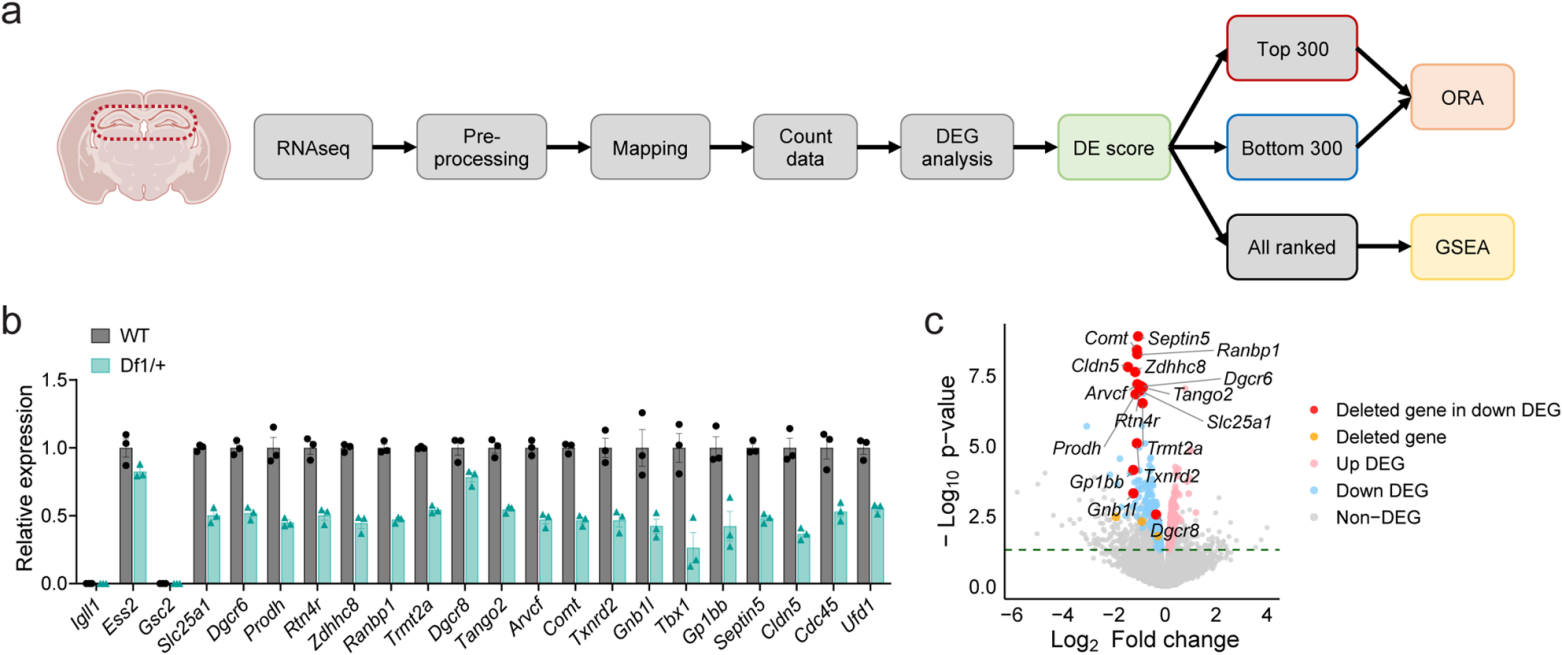
Transcriptomic profiling reveals distinct gene expression patterns in Df1/+ hippocampus. **a** Workflow of RNA sequencing analysis performed on the dorsal hippocampus of male Df1/+ mice. WT *n* = 3, Df1/+ *n* = 3. **b** Relative expression of genes within the deleted region in Df1/+ mice. **c** Volcano plot of genes passing the expression threshold (18,725 genes). Red dots represent differentially expressed genes (DEGs) located within the deleted region in Df1/+ mice. Orange dots indicate non-DEGs within the deleted region. Pink dots denote the top 300 upregulated DEGs, while blue dots indicate the bottom 300 downregulated DEGs. Grey dots represent all remaining genes. The green dotted line indicates the significance threshold (*p* = 0.05)

RNA-seq was performed on the dorsal hippocampal samples from WT and Df1/+ mice (Fig. 2a). Following normalization with the TMM method using the edgeR pipeline [25], a total of 41,079 genes were detected across all samples. As an internal validation of the data, we confirmed that 20 of the 22 genes located within the Df1 deletion interval [22] were expressed above the detection threshold, all of which showed reduced expression consistent with hemizygous gene dosage (Fig. 2b).

Differentially expressed genes (DEGs) were initially defined using a stringent statistical threshold of FDR-adjusted *p* < 0.05. However, this criterion yielded only a limited number of DEGs, insufficient for meaningful downstream pathway enrichment analysis (Fig. S2a, b). The 2-fold change cutoff (|log_2_FC| > 1) is widely used as a minimum effect-size filter in transcriptomic studies [26], including studies of brain disorders using either FDR-adjusted [27–29] or nominal p-values [30] in combination with this threshold. However, applying this conventional relaxed criterion (*p* < 0.05 & |log_2_FC| > 1.0) increased the number of DEGs but excluded approximately half of the deleted genes, with many of the remaining genes only marginally exceeding the fold-change boundary. Conversely, defining DEGs solely by *p* < 0.05 resulted in an excessively large DEG set, likely introducing substantial noise and complicating pathway-level interpretation.

To more reliably capture biologically meaningful transcriptional changes in Df1/+ mice, we implemented a custom Differential Expression (DE) score-based framework. First, to exclude lowly expressed genes with unreliable fold-change estimates [31–33], we applied a mild expression filter (a.value ≥ 0.5, corresponding to an average counts-per-million of ~ 1.4), retaining 18,725 genes. The DE score then integrates statistical significance, fold change, and expression abundance into a single continuous metric rather than applying a fold-change cutoff [34,35]. This framework is conceptually related to the previously proposed π-value method [36] and the signed significance metric for preranked GSEA [37], with the additional incorporation of expression abundance to prioritize robustly detected transcripts (Fig. S2c; see Methods for details). Genes were ranked by the DE score, and DEGs for ORA were defined as the top 300 upregulated and bottom 300 downregulated genes among those with *p* < 0.05 (1,616 genes), using all 18,725 genes as the background (Fig. 2c). GSEA was additionally performed using the full ranked list of 18,725 genes.

To assess the robustness of this approach, three independent validation procedures were performed: stratified bootstrap resampling (500 iterations), subsampling validation (100 iterations), and permutation testing. Jaccard indices ranged from approximately 0.39–0.44, indicating moderate reproducibility given the small sample size, and permutation testing confirmed that the observed rankings substantially exceeded null expectations (empirical *p* = 0.0560; Fig. S2d, e; Table S2-2). Multivariate analyses (MANOVA λ = 0.0554, *p* = 0.0130; PERMANOVA R² = 0.7177, *p* = 0.1000; ANOSIM *R* = 1.000, *p* = 0.1000) yielded large effect sizes consistent with genotype-driven separation, with the p-values reflecting the limited number of possible permutations (*n* = 3 per group). Principal component analysis (PCA) revealed a concordant pattern, with PC1 (75.4%) and PC2 (9.7%) capturing the majority of between-genotypes variance (Fig. S2f; Table S2-2). Notably, this criterion captured all 24 genes defined as DEGs by FDR *q* < 0.05 (Fig. S2b), suggesting that the DE score approach retains more statistically significant genes than p-value and fold-change-based criteria. Also, 16 out of 20 detected genes within the Df1 deletion interval, including *Comt* (Catechol-O-methyltransferase), *Cldn5* (Claudin 5), and *Septin5*, were captured among the downregulated DEGs (Fig. S2b).

### Over-representation and gene set enrichment analyses reveal functional implications of transcriptomic changes

To investigate the functional implications of the top and bottom 300 DEGs, we performed Gene Ontology (GO) enrichment analysis focusing on Biological Process (BP) terms using g: Profiler followed by semantic similarity clustering with GOSemSim.

**Fig. 3:**
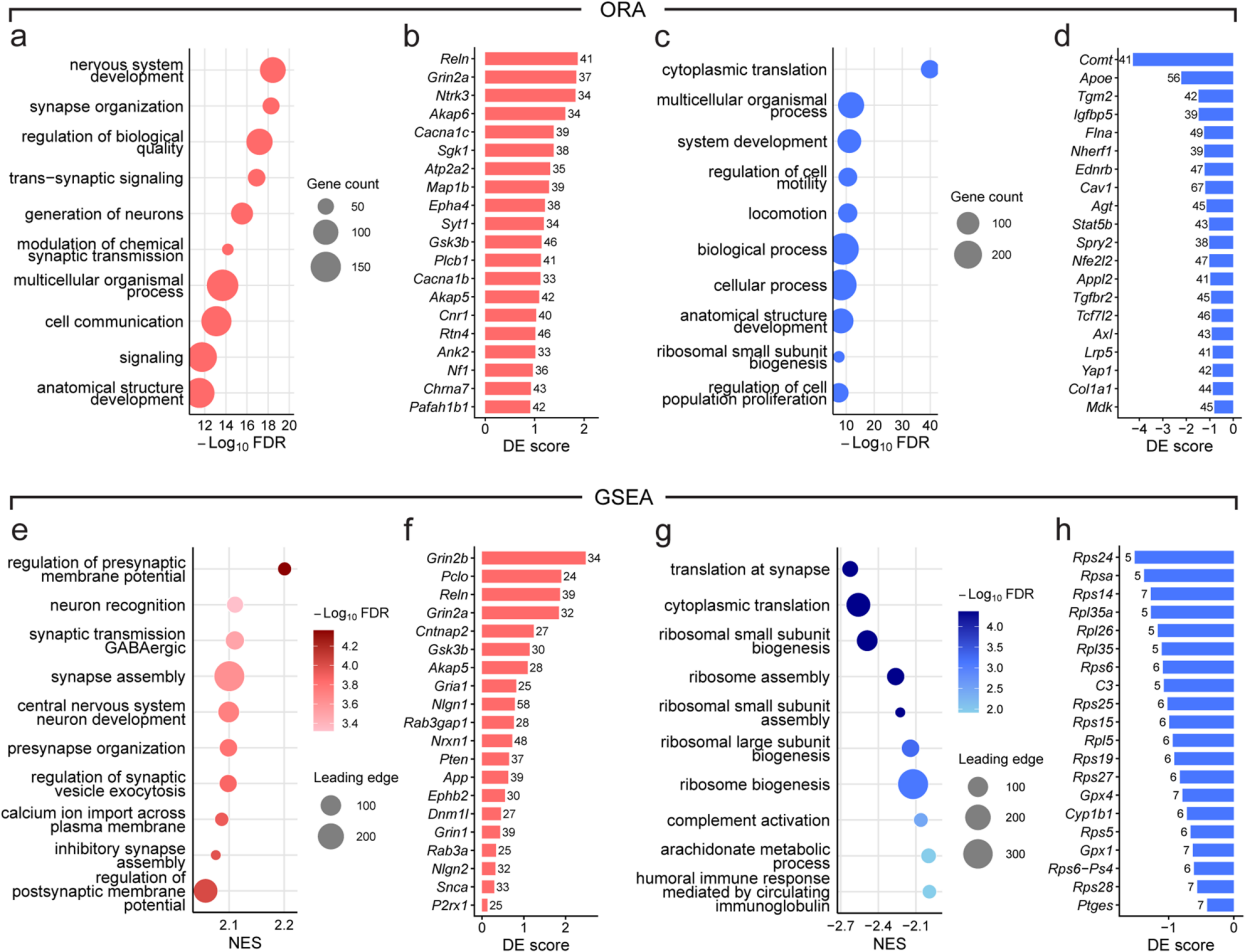
Over-representation and gene set enrichment analyses reveal functional implications of transcriptomic changes. **a**, **c** Top 10 enriched Gene Ontology Biological Process (GO: BP) terms identified by over-representation analysis (ORA) for upregulated (**a**) and downregulated DEGs (**c**). Dot size reflects the number of genes associated with each term. **b**, **d** ORA-based top 20 hub genes for significantly upregulated (**b**) and downregulated pathways (**d**). Genes are ranked by term count (number of representative terms in which each gene appears), with ties broken by absolute DE score. Bar length represents the DE score, and the number at the end of each bar indicates the term count. **e**, **g** Top 10 positively (**e**) and negatively (**g**) enriched GO: BP terms identified by gene set enrichment analysis (GSEA). The x-axis represents normalized enrichment score (NES). Dot size represents the number of leading edge genes for each term and the dot color reflects statistical significance. **f**, **h** GSEA-based top 20 hub genes for upregulated (**f**) and downregulated pathways (**h**). Genes are ranked by term count (number of pathways in which each gene appears as a leading-edge member), with ties broken by absolute DE score. Bar length represents the DE score, and the number at the end of each bar indicates the term count. Full data are provided in Additional file 4

The top 300 upregulated DEGs were predominantly enriched in neuronal and synaptic pathways (Fig. 3a). The most significantly enriched terms included “nervous system development”, “synapse organization”, “trans-synaptic signaling”, and “modulation of chemical synaptic transmission”. To identify hub genes driving the functional enrichment patterns, we examined the representation of each DEG across the significantly upregulated terms. The most represented hub genes included established risk genes for schizophrenia and other neurodevelopmental disorders, such as *Reln* (Reelin), *Grin2a* (NMDA receptor subunit 2A), *Cacna1c* (Calcium voltage-gated channel subunit alpha1 C), *Gsk3b* (Glycogen synthase kinase 3 beta), *Chrna7* (Cholinergic receptor nicotinic alpha 7 subunit) and *Pafah1b1* (Platelet-activating factor acetylhydrolase IB subunit alpha, also known as LIS1; Fig. 3b) [38–41]. These results indicate that the upregulated DEGs converge on synaptic function and include multiple genes previously implicated in psychiatric disorders, including schizophrenia.

The bottom 300 downregulated DEGs were strongly enriched for pathways related to protein synthesis and developmental regulation (Fig. 3c). The most prominent term was “cytoplasmic translation”, encompassing numerous ribosomal protein genes (e.g., *Rpsa*,* Rpl11*,* Rps24*), suggesting a reduction in translational capacity (Additional file 4, Table S3-4). Additional enriched terms included “ribosomal small subunit biogenesis”, “regulation of cell motility”, and “regulation of cell population proliferation”, indicating involvement of downregulated genes in both ribosomal processes and broader cellular regulation. Several hub genes have been implicated in neurodevelopmental and psychiatric disorders. These included *Comt*, one of the hemizygously deleted genes in Df1/+ mice, whose low-activity allele has been identified as a risk factor for cognitive decline and psychosis in 22q11.2DS [42], as well as *Apoe* (Apolipoprotein E), a major genetic determinant of cognitive function and neurodegeneration [43], *Cav1* (Caveolin 1), *Tgfbr2* (Transforming growth factor beta receptor 2), and *Tcf7l2* (Transcription factor 7-like 2), a downstream effector of the Wnt/β-catenin signaling pathway associated with susceptibility to schizophrenia and autism spectrum disorder [44] (Fig. 3d).

Collectively, upregulated DEGs were predominantly involved in synaptic connectivity and signaling, whereas downregulated DEGs converged on translational machinery and cell proliferation pathways.

To assess the robustness of these findings, we compared the enrichment results from DE score-based top/bottom 300 DEGs with those obtained using conventional thresholds: FDR < 0.05 (24 DEGs) and adjusted *p* < 0.05 and |log2FC| > 1.0 (381 DEGs). For upregulated genes, neither conventional approach yielded any significantly enriched GO: BP terms, in contrast to the 601 terms detected by the DE score method (Fig. S3a, b). For downregulated genes, The FDR < 0.05 threshold (21 downregulated genes) yielded 163 enriched terms, sharing 72 terms with the DE score approach; however, these terms were largely driven by the hemizygous deletion-interval genes and dominated by immune and complement-related pathways (Fig. S3a, b). The adjusted *p* < 0.05 and |log2FC| > 1.0 threshold yielded 65 enriched terms, with only 28 shared with the 581 DE score terms, predominantly broad developmental processes such as “anatomical structure development” and “animal organ development” (Fig. S3c, d). Notably, the top enriched terms unique to the DE score approach included “cytoplasmic translation”, “translation at synapse”, and “translation at postsynapse”, all of which were absent from both conventional threshold results despite all constituent genes being individually significant (*p* < 0.05), because their log2 fold-changes were modest. In contrast, the top terms detected exclusively by the adjusted *p* < 0.05 and |log2FC| > 1.0 approach were driven by a small number of genes with large fold-changes but low expression levels and were predominantly related to kidney development, a finding with limited relevance to the hippocampal context of this study. These results indicate that the DE score approach, by integrating effect size and statistical significance along a continuous spectrum, captures coordinated but individually modest expression changes in neuronal and synaptic pathways that fall below conventional fold-change thresholds.

GSEA revealed significant alterations across multiple neuronal and synaptic pathways. The positively enriched terms included “regulation of pre/postsynaptic membrane potential”, “GABAergic synaptic transmission”, and “synapse assembly” (Fig. 3e). The most recurrent hub genes across the significantly enriched terms included NMDA receptor subunits (*Grin1*, *Grin2a*, *Grin2b*), *Reln*, *Cntnap2* (Contactin-associated protein-like 2), and synaptic adhesion molecules (*Nlgn1*; Neuroligin 1, *Nrxn1*; Neurexin 1; Fig. 3f) [45, 46].

In contrast, several pathways associated with translational machinery were negatively enriched, including “translation at synapse”, “cytoplasmic translation”, “ribosomal small/large subunit biogenesis”, and “ribosome assembly”, indicating broad downregulation of protein synthesis processes (Fig. 3g). Both small ribosomal subunit genes and large ribosomal subunit genes appeared repeatedly among these negatively enriched terms (Fig. 3h). In addition, immune-related pathways, such as “complement activation”, and “humoral immune response mediated by circulating immunoglobulin”, also showed negative enrichment. Among the hub genes, *C3* (Complement component 3), a central component of the complement cascade implicated in microglia-mediated synaptic pruning [47, 48], and modulators of inflammatory signaling, such as *Gpx4*, *Gpx1* (Glutathione peroxidase 4 and 1), and *Ptges* (Prostaglandin E synthase), were represented.

Together, the enrichment profile indicates upregulation of genes involved in synaptic signaling and neuronal connectivity, coupled with a downregulation of translational and immune-related pathways. These results provide a comprehensive overview of molecular programs potentially contributing to synaptic and network-level changes.

To further contextualize these pathway-level changes, we evaluated the cellular origin of the transcriptomic alterations by performing GSEA using cell type-specific gene sets curated from PanglaoDB [49]. The upregulated genes were enriched in neuronal populations, particularly interneurons, GABAergic neurons, and dopaminergic neurons (Fig. S4a, b), whereas downregulated genes were enriched in glial populations, including microglia, astrocytes, and oligodendrocytes (Fig. S4c, d). Complementary deconvolution analysis using MuSiC [50] with single-cell hippocampal reference data (Allen Brain Atlas) indicated that overall cell type proportions were generally unchanged (Fig. S4e), suggesting that these enrichments primarily reflect cell type-specific transcriptional regulation rather than shifts in cellular composition. Collectively, these analyses indicated cell type changes toward enhanced neuronal signaling and reduced glial transcriptional programs in the hippocampus of Df1/+ mice.

### Integrated analysis reveals synaptic upregulation and translational downregulation

**Fig. 4:**
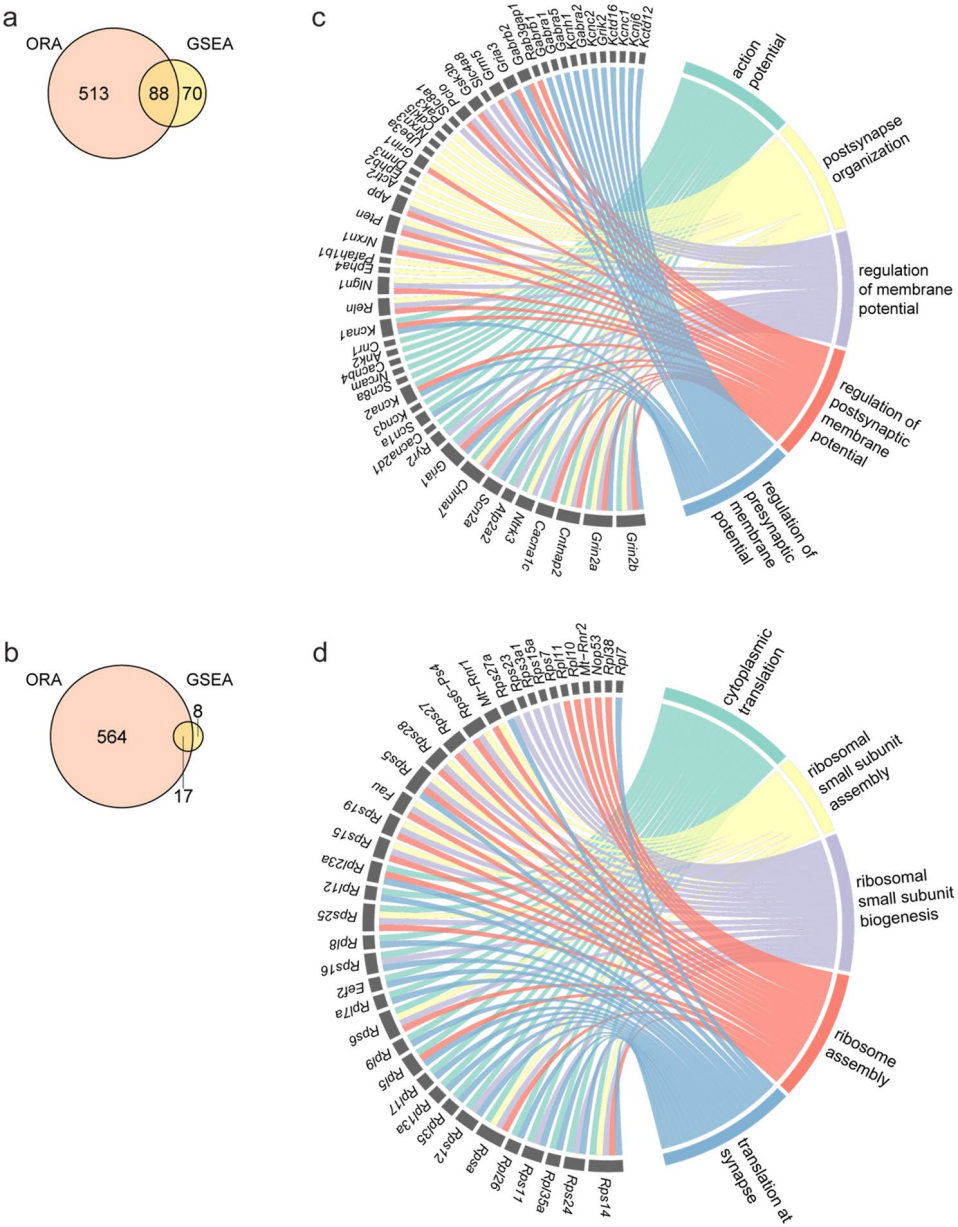
Integrated analysis reveals synaptic upregulation and translational downregulation. **a**, **b** Venn diagrams showing the number of enriched pathways identified by ORA and GSEA and their overlap for upregulated terms (**a**) and downregulated terms (**b**). **c**, **d** Chord plots illustrating the top 5 concordant GO: BP terms with the higher combined scores and their top 20 contributing genes with the higher priority scores for upregulated (**c**) and downregulated pathways (**d**). Full data are provided in Additional file 4

ORA identified 601 upregulated and 581 downregulated significant pathways, while GSEA detected 158 upregulated and 25 downregulated significant pathways. Integration of both analyses identified 88 upregulated and 17 downregulated common pathways (Fig. 4a, b). To prioritize pathways showing strong and concordant signals in both ORA and GSEA, we created a combined score that reflects how strongly each pathway is supported by both ORA and GSEA.

The top upregulated pathways with the higher combined scores included “action potential”, “postsynapse organization”, and “regulation of membrane potential” (Fig. 4c). The “action potential” pathway included glutamate receptors (*Grin2a*,* Grin2b*,* Gria1*: Glutamate ionotropic receptor AMPA type subunit 1), voltage-gated channels (*Scn1a*: Sodium voltage-gated channel alpha subunit 1, *Cacna1c*), and synaptic regulators (*Reln*,* Ntrk3*: Neurotrophic receptor tyrosine kinase 3) [41, 51–53]. The “postsynapse organization” pathway highlighted synaptic adhesion molecules (*Nlgn1*,* Nrxn1*) and scaffolding proteins (*Ephb2*: Ephrin type-B receptor 2, *App*: Amyloid beta precursor protein, *Ube3a*: Ubiquitin protein ligase E3A) [46,54–56].

Conversely, pathways associated with protein synthesis were significantly downregulated (Fig. 4d), including “cytoplasmic translation”, “ribosomal small subunit assembly/biogenesis”, and “translation at synapse”. Representative ribosomal genes (*Rpsa*, *Rps14*,* Rps6*,* Rpl35*,* Rpl26*) were commonly downregulated across these terms, reflecting broad suppression of translational machinery.

Altogether, these findings demonstrate transcriptional changes characterized by upregulation of genes promoting neuronal excitability, synaptic organization, and membrane potential regulation, coupled with downregulation of translational components. The concordance between ORA and GSEA further supports the robustness of these pathway-level changes.

### Cross-species comparison with human schizophrenia hippocampal transcriptomes

To evaluate cross-species correspondence of gene expression changes, we compared the hippocampal transcriptome of the Df1/+ mice with postmortem human schizophrenia hippocampal samples (GSE138082) [23]. Following preprocessing, 77 human samples (39 controls, 38 schizophrenia) with 29,384 genes passing expression filtering (a.value ≥ 0.5) were retained for analysis. DE scores were calculated for human dataset and top and bottom 300 genes within 2,467 genes passing *p* < 0.05 were defined as DEGs (Fig. S5a-c). Notably, genes within the 22q11.2 region that were strongly downregulated in the Df1/+ mouse (e.g., *Comt*, *Septin5*, *Ranbp1*: RAN binding protein 1) showed no significant differential expression in the human dataset (Fig. S5a), consistent with the human cohort being composed of idiopathic schizophrenia cases in which 22q11.2 deletion carriers are absent or extremely rare. Individual-level genetic screening data, including 22q11.2 deletion status, were not reported for this cohort [23], and confirmation was not possible from the publicly available metadata.

**Fig. 5:**
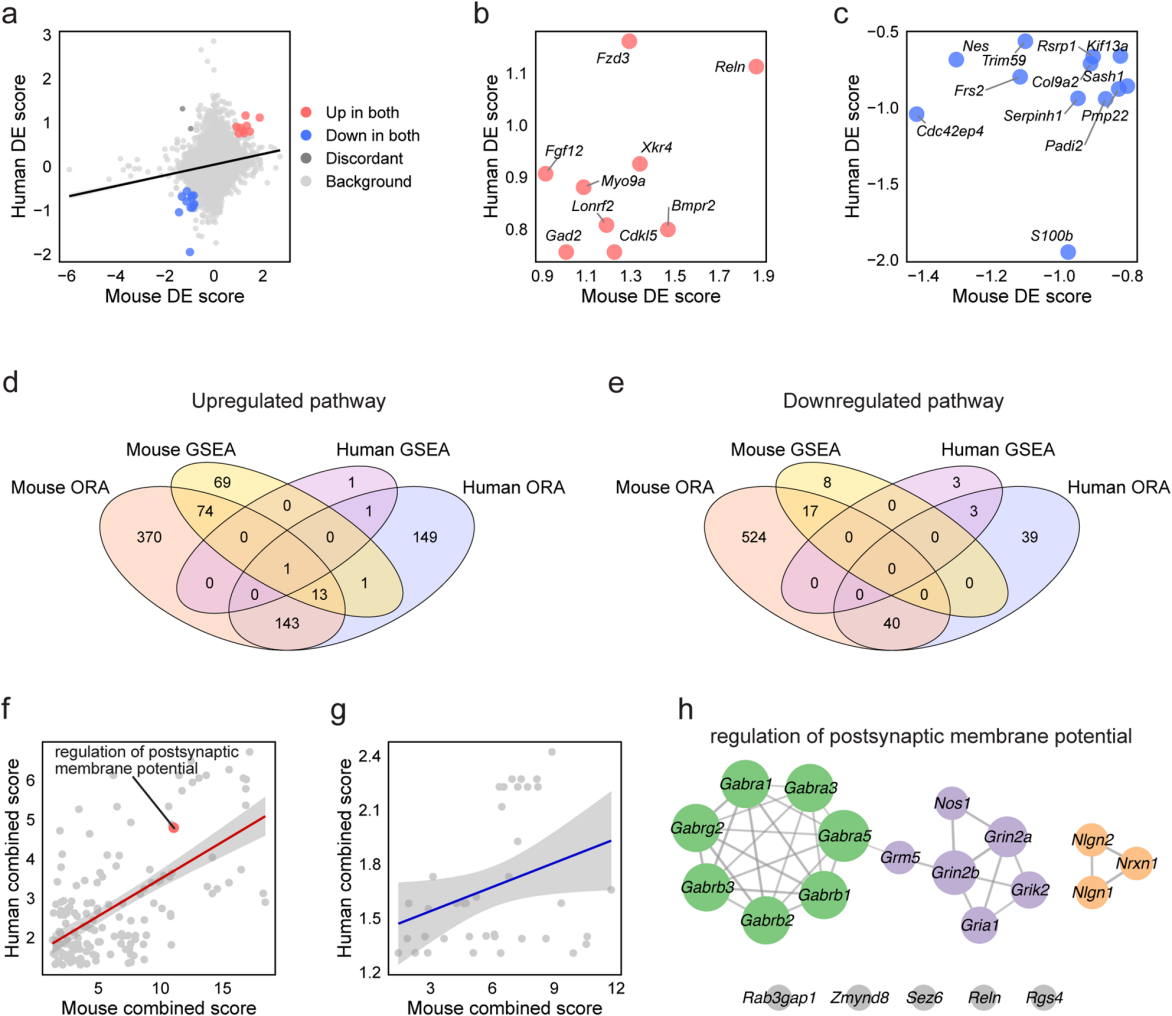
Cross-species transcriptomic comparison between mouse and human hippocampus. **a** Scatter plot showing shared genes between mouse and human (13,965 genes). Genes upregulated in both species are shown in red, and genes downregulated in both species are shown in blue. Genes with discordant directionality are shown in dark grey, and all other genes are shown in light grey. **b**, **c** Scatter plots showing the common upregulated (**b**) and common downregulated DEGs (**c**) between species. **d**, **e** Venn diagrams showing the number of enriched GO: BP pathways identified by ORA and GSEA in both species and their overlaps for upregulated (**d**) and downregulated pathways (**e**). **f**, **g** Scatter plots showing shared pathways identified by ORA and/or GSEA between species for upregulated (**f**) and downregulated pathways (**g**). A pathway identified across both species and detected by both analyses is highlighted in red (for upregulated pathway). **h** Protein-protein interaction network of genes within the “regulation of postsynaptic membrane potential” pathway, the only pathway identified across both species by both ORA and GSEA. Node size represents the number of connections (STRING degree), edge width reflects STRING interaction confidence scores, and node colors indicate functional categories (green: GABA receptors; purple: glutamatergic signaling components; orange: synaptic adhesion molecules; grey: other regulatory proteins). Full data and statistical results are provided in Additional file 6 and Table S2-3

One-to-one ortholog mapping identified 13,965 shared genes between species. Spearman’s rank correlation of DE scores revealed a modest but highly significant positive correlation (ρ = 0.199, *p* < 0.0001), indicating a degree of cross-species correspondence in transcriptional changes (Fig. 5a, Table S2-3). Among 23 genes differentially expressed in both species, 21 showed concordant regulation (9 upregulated, 12 downregulated), although the overall overlap did not reach statistical significance (Fisher’s exact test, odds ratio = 1.48, *p* = 0.0911).

The concordantly upregulated DEGs included *Reln*, *Gad2* (Glutamate decarboxylase 2), *Fzd3* (Frizzled class receptor 3), *Bmpr2* (Bone morphogenetic protein receptor type 2), and *Cdkl5* (Cyclin-dependent kinase-like 5; Fig. 5b). Among these, *Reln* is involved in neuronal migration and synaptic plasticity [57], *Gad2* in GABAergic neurotransmission [58], and *FZD3* has been associated with schizophrenia susceptibility [59, 60]. The concordantly downregulated DEGs included glial markers (*Nes*: Nestin, *S100b*: S100 calcium binding protein B, *Padi2*: Peptidyl arginine deiminase 2), extracellular matrix components (*Col9a2*: Collagen type IX alpha 2 chain, *Serpinh1*: Serpin family H member 1), and myelination-associated genes (*Pmp22*: Peripheral myelin protein 22; Fig. 5c). While the gene-level overlap was limited, the high concordance rate (91.3%) among shared DEGs spanned both synapse- and glia-related functional categories.

To evaluate pathway-level overlap, we integrated ORA and GSEA results from both species. In humans, ORA identified 308 upregulated and 82 downregulated pathways (Fig. S5d-g), while GSEA detected 3 upregulated and 6 downregulated pathways (Fig. S5h-k), yielding two upregulated and three downregulated consensus pathways (Fig. 5d, e). Spearman’s correlation of the combined scores between species revealed significant pathway-level concordance for upregulated pathways (Fig. 5f; *p* < 0.0001) and downregulated pathways (Fig. 5g; *p* = 0.0341). “Regulation of postsynaptic membrane potential” was the only pathway identified as upregulated across both species by both ORA and GSEA. Shared genes within this pathway included GABA receptor subunits (*Gabra1*,* Gabra3*,* Gabra5*,* Gabrb1*,* Gabrb2*,* Gabrb3*,* Gabrg2*), glutamate receptor subunits (*Gria1*,* Grin2a*,* Grin2b*,* Grik2*: Glutamate ionotropic receptor kainate type subunit 2), synaptic adhesion molecules (*Nrxn1*,* Nlgn1*,* Nlgn2*: Neuroligin 2)^46^, and key regulators including *Reln* and *Nos1* (Nitric oxide synthase 1), a component of the NMDA receptor-PSD-95 postsynaptic complex and a schizophrenia susceptibility gene [61] (Fig. 5h).

Altogether, the cross-species comparison revealed limited but directionally consistent gene-level overlap and a significant pathway-level correspondence centered on postsynaptic membrane potential regulation. These findings suggest that the Df1/+ hippocampal transcriptome partially recapitulates molecular features observed in human schizophrenia, particularly in excitatory–inhibitory synaptic signaling, while also highlighting species-specific differences that constrain direct translational inference.

## Discussion

The present study provides a comprehensive transcriptomic characterization of hippocampal alterations in Df1/+ mice, a model of 22q11.2DS. Consistent with previous reports, Df1/+ mice exhibited impaired contextual fear memory without gross locomotor deficits, along with enhanced sensitivity to psychostimulant challenge [14, 24]. RNA-seq analysis revealed a transcriptional landscape characterized by upregulation of genes involved in synaptic signaling, neuronal connectivity, and glutamatergic and GABAergic neurotransmission, accompanied by downregulation of genes related to translational machinery. Importantly, cross-species comparison with postmortem human schizophrenia hippocampal samples demonstrated overlap in key molecular signatures, particularly in pathways regulating postsynaptic membrane potential and in genes related to glial function. These findings provide mechanistic insight into hippocampal dysfunction in 22q11.2DS and its relevance to psychiatric disorders.

### Transcriptomic alterations in Df1/+ hippocampus: E/I imbalance and impaired cellular homeostasis

The most prominent transcriptomic alteration in the Df1/+ hippocampus was the enhanced expression of genes involved in synaptic signaling and neurotransmission, suggesting fundamental disruption of excitatory-inhibitory (E/I) balance. This molecular pattern aligns with the E/I imbalance hypothesis of schizophrenia, which proposes that cognitive deficits and psychotic symptoms arise from disrupted coordination between excitatory and inhibitory circuits [62, 63]. Our analysis revealed significant upregulation of multiple glutamate receptor subunits (*Grin1*, *Grin2a*, *Grin2b*, *Gria1*) and voltage-gated ion channels (*Scn1a*, *Cacna1c*), alongside GSEA enrichment of “GABAergic synaptic transmission”. Cell-type enrichment analysis showed that upregulated genes were preferentially expressed in interneurons and GABAergic neurons (Fig. S3a, b). Both glutamatergic and GABAergic dysfunction have been extensively documented across 22q11.2DS models [17, 64–72], and magnetic resonance spectroscopy studies in human patients indicate that individuals with 22q11.2DS exhibit altered E/I balance, with disruptions in glutamatergic and GABAergic signaling associated with cognitive and psychotic symptoms [63, 73].

The upregulation of both excitatory and inhibitory synaptic components in our study may represent transcriptional changes associated with functional circuit alterations. Supporting this interpretation, pathway enrichment including “regulation of postsynaptic membrane potential” and “postsynapse organization”, alongside the concurrent upregulation of synaptic adhesion molecules (*Nlgn1*, *Nrxn1*, *Cntnap2*) and key synaptic regulators. Among these, the upregulation of *Reln* emerges as a notable finding, as Reelin modulates both inhibitory and excitatory synaptic transmission through regulation of receptor trafficking and synaptic plasticity [74, 75, 57, 76]. While most schizophrenia models and patients show Reelin reduction [77–79], the increased *Reln* mRNA in Df1/+ hippocampus could indicate altered transcriptional regulation responding to the changes in interneuron function and excitatory plasticity.

Among the downregulated genes, *Comt*, one of the genes deleted in Df1/+ mice, plays a critical role in dopamine metabolism and has been implicated in cognitive dysfunction and schizophrenia risk. Selective *Comt* overexpression has been shown to prevent MK-801-induced hyperlocomotion in Df1/+ mice [19], suggesting *Comt* expression change is possibly related to the behavioral alterations observed in the present study. The concurrent downregulation of genes involved in neuronal homeostasis, such as *Apoe* and *Cav1*, alongside immune-related pathways including complement activation, with hub genes such as *C3* and inflammatory modulators (*Gpx4*, *Gpx1*, *Ptges*), suggests broader alterations in cellular maintenance mechanisms. The downregulation of complement components may compromise microglia-mediated synaptic pruning and debris clearance [47, 48], processes relevant to the cognitive and behavioral deficits in 22q11.2DS.

### Cross-species correspondence and translational relevance

The cross-species correspondence of transcriptomic alterations between Df1/+ mice and human schizophrenia hippocampal samples provides translational context for the 22q11.2DS model and highlights shared molecular pathways underlying hippocampal dysfunction. At the gene level, the concordant upregulation of *Reln* and *Gad2* in both species may reflect transcriptional patterns associated with GABAergic interneuron dysfunction, a core pathophysiological feature of schizophrenia proposed to underlie cognitive deficits, negative symptoms, and sensory processing abnormalities [80]. Among the concordantly downregulated DEGs, glial markers, extracellular matrix components, and myelination-associated genes were represented, aligning with neuropathological evidence of altered glial density and white matter abnormalities in schizophrenia [81, 82] and 22q11.2DS patients [83, 84]. This is consistent with findings that *Tbx1* haploinsufficiency, a gene within the deleted region, leads to fimbria myelination deficits correlating with reduced cognitive processing speed [18]. While the gene-level overlap was limited, the high concordance rate among shared DEGs and the involvement of both synapse- and glia-related functional categories suggest that similar biological processes are affected in both species.

At the pathway level, cross-species comparison revealed notable correspondence of molecular signatures. The shared upregulation of the “regulation of postsynaptic membrane potential” pathway across species included both glutamatergic receptor subunits (*Grin2a*, *Grin2b*, *Gria1*, *Grik2*) and GABAergic receptor subunits (*Gabra1*, *Gabra3*, *Gabra5*, *Gabrb1*, *Gabrb2*, *Gabrb3*, *Gabrg2*), alongside synaptic adhesion molecules (*Nrxn1*, *Nlgn1*, *Nlgn2*) and key regulators including *Reln*, and *Nos1*. This overlap between a genetic mouse model and idiopathic human schizophrenia is consistent with disrupted E/I balance as a shared mechanism underlying hippocampal dysfunction, in line with recent neuroimaging findings in 22q11.2DS patients [63]. The pathway enrichment demonstrates that both excitatory and inhibitory neurotransmission systems are concordantly affected across species, reinforcing the E/I imbalance hypothesis as a unifying framework for understanding schizophrenia pathophysiology [63,85]. However, the limited number of shared pathways detected by GSEA in the human dataset and the modest overall gene-level correlation indicate that the degree of cross-species overlap should not be overstated, and species-specific differences likely contribute substantially to the transcriptomic landscape in each system.

Notably, the enrichment of autism risk genes within the shared upregulated pathway (*Reln*, *Cntnap2*, *Nrxn1*, *Grin2b*, *Gria1*, *Nlgn1*) [86] suggests that the molecular overlap observed here may extend beyond schizophrenia. E/I imbalance has also been proposed as a unifying mechanism in autism spectrum disorder [87], supporting the view that 22q11.2DS represents a shared etiological axis across neurodevelopmental psychiatric disorders [3, 88]. This cross-disorder molecular overlap highlights the potential for shared therapeutic strategies targeting E/I balance across multiple neuropsychiatric conditions.

### Limitations

Several limitations should be considered when interpreting our findings. First, the relatively small sample size (*n* = 3 per group) for RNA-seq analysis, while appropriate for exploratory transcriptomic profiling and consistent with previous studies in this field, limits statistical power for detecting subtle expression changes. Although we employed rigorous validation procedures including bootstrap resampling, subsampling, and permutation testing to assess reproducibility, and multivariate analyses confirmed robust genotype separation, independent validation using larger cohorts and orthogonal methods such as qPCR or protein-level analyses would strengthen these findings. Second, the mouse RNA-seq data were obtained exclusively from male Df1/+ and WT animals, precluding examination of sex-specific transcriptomic effects. In the human dataset, predominance of male subjects and the limited female sample size preclude formal sex-stratified analysis and limit generalizability to female patients. Future studies with larger, sex-balanced cohorts are warranted to evaluate potential sex-specific molecular signatures. Third, our study is cross-sectional and observational, preventing determination of causal relationships or developmental trajectories of the observed molecular changes. The synaptic gene upregulation remains inferential without functional validation or intervention studies.

## Conclusion

In summary, our comprehensive transcriptomic analysis of the Df1/+ mouse hippocampus reveals alterations in synaptic signaling, translational machinery, and glial function that overlap with those observed in human schizophrenia. The upregulation of genes involved in GABAergic and glutamatergic neurotransmission, synaptic organization, and neuronal connectivity suggests a transcriptional reorganization accompanying the circuit dysfunction driven by E/I imbalance, while the downregulation of ribosomal proteins and glial markers indicates impaired protein homeostasis and reduced glial support. The molecular signatures shared across species support the translational relevance of the 22q11.2DS mouse model and provide a molecular framework for understanding hippocampal dysfunction in psychiatric disorders. This multilevel disruption, spanning synaptic function, translational capacity, and glial support, provides a mechanistic framework for understanding why behavioral and cognitive deficits persist in 22q11.2DS despite upregulation of synaptic genes.

## Methods

### Animals

Adult Df1/+ mice (backcrossed to C57BL/6J for > 10 generations) and wild-type (WT) littermates were used for all experiments. Mice were maintained under standard laboratory conditions (temperature 22 ± 2 °C; 12 h light/dark cycle, lights on at 08:00) with ad libitum access to food and water. All animal experiments were approved by the Animal Care and Use Committee of the Institute for Protein Research, The University of Osaka (protocol number: R04-01-2). Genotyping of Df1/*+* mice was performed by PCR using the following primers: Df1-ES2-tarF (Sense, common for WT & Mut; 5′- GCC TTT GTT TTC AAG CTC CA -3′), Df1-ES2-tarR (Anti-sense for Mut; 5′- TAC CGG TGG ATG TGG AAT GT -3′), and Df1-AS-W (Anti-sense for WT; 5′- ACT GTC GTC ATG TAT CTG ACC − 3′).

### Behavioral tests

All behavioral tests were conducted between 10:00 and 17:00. Mice were acclimated to the testing room for at least 30 min prior to testing. The apparatus for each test was cleaned with 70% ethanol between subjects to remove odor cues.

#### Fear conditioning test

A total of sixteen mice (8 Df1/+ and 8 WT, 5 males and 3 females for each), aged 13 weeks, were used. Fear conditioning was performed using a NIR Video Fear Conditioning System (Med Associates, Inc., VT, USA). The conditioning chamber (#VFC-008, Med Associates, Inc., VT, USA) equipped with a stainless-steel grid floor was housed in a sound-attenuating enclosure (#NIR-022MD, Med Associates, Inc., VT, USA).

Day 1 (Conditioning): Each mouse was placed in the chamber and allowed to explore for 120 s, followed by three pairings of a conditioned stimulus (CS; 20 s white noise, 80 dB, 6000 Hz) with an unconditioned stimulus (US; 0.6 mA foot shock delivered during the last 2 s of the CS). Intertrial intervals (ITI) were 90 s and 150 s. Thirty seconds after the final CS–US pairing, mice were returned to their home cages.

Day 2 (Testing): For the contextual test, mice were re-exposed to the original chamber for 300 s without any stimulus presentation. Immediately afterward, the cued test was performed in a novel context (black Plexiglas triangular roof, white Plexiglas floor, vanilla odor). Mice were habituated to the altered chamber for 120 s, followed by the same 20 s CS presentations four times. Interstimulus intervals were 150 s, 90 s, and 120 s. Freezing behavior was automatically recorded using VideoFreeze software (v3.02, Med Associates, Inc., VT, USA) with the following parameters: sample rate = 30 Hz, motion threshold = 18, detection method = linear, and minimum freeze duration = 30 frames (1 s).

#### Open field test

Fifteen mice (7 Df1/+, 4 males and 3 females; 8 WT, 3 males and 5 females), aged 10–13 weeks, were tested in a square open-field chamber (#ENV-515-16, Med Associates, Inc., VT, USA) equipped with a grid floor. Locomotor activity was monitored for 30 min using Activity Monitor software (v6.02, Med Associates, Inc., VT, USA). The apparatus contained infrared beams on all sides to track mouse movement, and the illumination level in the chamber was maintained at 230–240 lx. Total distance traveled was used as an index of locomotor activity. The arena was subdivided into a 16 × 16 grid along the x- and y-axes, and the central zone was defined as blocks 3.5–13 on both axes. Time spent in the central zone was calculated as a measure of anxiety-related behavior.

#### Acute psychostimulant response

The same cohort of mice used for the open field test was used to assess locomotor response to a psychostimulant at 12–16 weeks of age. Mice were intraperitoneally administered 0.2 mg/kg (+)-MK-801 hydrogen maleate (MK-801; #M107, Sigma-Aldrich, MO, USA), a dose previously shown to induce robust hyperlocomotion in C57BL/6J mice [89–91], and placed in the center of a circular arena (41-cm diameter; 40-cm wall height) for 30 min of free exploration. Locomotor activity was recorded using an overhead camera, and the distance traveled was quantified using EthoVision XT (Noldus Information Technology, Wageningen, The Netherlands). The illumination level at the center of the arena was approximately 30 lx.

#### Statistical analysis for behavioral data

All behavioral data were analyzed in GraphPad Prism 9 (RRID: SCR_002798; GraphPad Software Inc., CA, USA). Data are presented as mean ± SEM. Normality of data distribution was checked by the Shapiro-Wilk test. Between-group comparisons were performed using unpaired *t*-tests or two-way analysis of variance (ANOVA), as appropriate. Within-group effects were analyzed by repeated measures ANOVA. When ANOVA showed significant main or interaction effects, post hoc comparisons were conducted using Bonferroni correction. Statistical significance was assessed using an α value of 0.05. Raw data and full statistical results are provided in Additional file 2 and Additional file 3 (Table S2-1).

### RNA sequencing (RNA-seq) and gene expression analysis

Samples were collected and prepared as a previous report [92]. Three male Df1/+ mice and three male WT littermates (12 weeks old) from a separate, behaviorally naïve cohort maintained under basal conditions were euthanized by decapitation. Brains were rapidly removed and sectioned coronally (1 mm thickness) using a mouse brain matrix (#MBS-S1C; BrainScience Idea Co., Ltd., Osaka, Japan). The dorsal hippocampus (dHipp) was dissected bilaterally according to the mouse brain atlas. Tissue samples were immersed in 150 µL RNAlater (#AM7020, Thermo Fisher Scientific, MA, USA), stored at 4 °C overnight, and then transferred to − 80 °C until RNA extraction. Total RNA was isolated using the RNeasy Plus Mini Kit (#74136, QIAGEN, Venlo, The Netherlands) following the manufacturer’s protocol.

RNA-seq libraries were prepared with the TruSeq Stranded mRNA Library Prep Kit (#20020595, Illumina, San Diego, CA, USA) and sequenced on an Illumina NovaSeq 6000 platform (RRID: SCR_016387) in 101 bp single-end mode (~ 10 million reads per sample). Reads were aligned to the mouse reference genome (NCBI RefSeq GCF_000001635.27_GRCm39) using STAR [93] (v2.7.11b), and gene-level quantification was performed using RSEM [94] (v1.2.28). Count normalization was performed by the trimmed mean of M-values (TMM) method, and differential expression analysis was carried out using edgeR [25] implemented in TCC-GUI [95] (iteration = 3; false discovery rate (FDR) cutoff = 0.1; elimination threshold = 0.05; low-count genes = not filtered).

Conventional approaches to DEG definition using fixed statistical and fold-change thresholds proved inadequate for this dataset (see Results for detailed evaluation). In particular, the commonly used criterion of *p* < 0.05 and |log_2_FC| > 1.0 excluded 8 of the 20 evaluable deletion-interval genes, including genes with FDR-adjusted *q* < 0.05, solely because their fold changes fell below the arbitrary 2-fold threshold. This is consistent with the broader observation that fixed fold-change cutoffs can substantially alter the biological interpretation of transcriptomic data [34] and with prior microarray studies of the Df1/+ mouse showing that deletion-interval genes typically exhibit 30–40% reductions in expression [21, 22, 96].

To address two complementary problems identified during this evaluation, we implemented a DE score-based framework incorporating both expression filtering and continuous abundance weighting. First, to address the inclusion of lowly expressed genes with artificially inflated fold changes, a recognized source of false positives in differential expression analysis [33], we applied a mild expression filter (a.value ≥ 0.5, corresponding to an average counts-per-million of ~ 1.4). This retained 18,725 genes (45.6% of all detected genes) for downstream analysis while excluding those with insufficient expression for reliable fold-change estimation. Second, to address the converse problem, that highly expressed genes with modest but biologically genuine fold changes are systematically excluded by hard fold-change cutoffs, we converted statistical significance, fold change, and expression abundance into a single continuous metric rather than imposing a binary threshold. It has been argued that hard fold-change cutoffs introduce bias [34] and should be replaced by formal integration of effect size into the test statistic [35]. Following this rationale, we developed a composite Differential Expression (DE) score, calculated for each gene as:

$$\begin{aligned} DE~score~&=~sign\left( {lo{g_2}FC} \right)~ \times ~\left( { - lo{g_{10}}\left( {p - value} \right)} \right)~ \\&\quad \times ~scaled~average~expression \end{aligned}$$

where log_2_FC represents the log_2_ fold change (m.value), *p*-value is the statistical significance from edgeR differential expression testing, and scaled expression is the normalized average expression level (a.value) scaled to the range [0, 1]. The rationale for this composite score is following: the sign(log_2_FC) preserves directionality of change, -log₁₀(p-value) weights genes by statistical confidence, addressing the “large fold change, large variance” issue [36], and scaled expression down-weights low-expression genes that may have unreliable fold change estimates [97].

Genes were ranked by their DE score. From the subset passing nominal significance (*p* < 0.05; *n* = 1,616), the top 300 upregulated and bottom 300 downregulated genes were defined as differentially expressed genes (DEGs) for over-representation analysis (ORA), using all 18,725 genes that passed the expression threshold as the background. The full ranked gene list was used as input for pre-ranked gene set enrichment analysis (GSEA). All results are collected in Additional file 4 (TMM normalized gene counts: Table S3-1, DEG list: Table S3-2).

To assess reproducibility under small-sample conditions, we applied stratified bootstrap resampling (*n* = 500), leave-one-out subsampling (*n* = 100), and permutation testing (*n* = 1000). For each resampling procedure, Jaccard indices between top/bottom 300 gene sets derived from resampled versus full datasets were calculated. Random seed was fixed at 123 to ensure full reproducibility. Also, principal component analysis (PCA) was performed on TMM-normalized counts of the top and bottom 300 genes, and group separation was evaluated statistically using permutational multivariate analysis of variance (PERMANOVA), and analysis of similarities (ANOSIM). For both PERMANOVA and ANOSIM, all possible permutations (< 999) were enumerated (*n* = 719), and therefore p-values correspond to complete permutation space. Full statistical results are provided in Additional file 3 (Table S2-2).

### Pathway enrichment analyses

#### Over-representation analysis (ORA)

Top 300 upregulated and bottom 300 downregulated genes were analyzed using g: Profiler [98] (https://biit.cs.ut.ee/gprofiler/gost; accessed on 3rd February 2026) with *Mus musculus* background and FDR *q* < 0.05. Gene Ontology Biological Process (GO: BP) terms [99, 100] were clustered based on semantic similarity using the R package GOSemSim [101] (v2.30.2; Wang method; complete linkage; cut height = 0.7), and representative terms with the smallest FDR *q*-values were retained (Additional file 4: Table S3-3, S3-4). To identify hub genes, we counted how many representative GO: BP terms each gene appeared in (term count). Genes were ranked primarily by term count in descending order, with ties broken by the absolute value of the DE score. The top 20 genes from each direction (upregulated and downregulated) were designated as hub genes, representing key molecular players most broadly involved across the altered biological networks.

#### Gene set enrichment analysis (GSEA)

GO: BP gene sets were obtained from MSigDB v2025.1 (m5.go.bp.v2025.1.Mm.symbols.gmt) [100, 102] and remapped using the official mouse chip file. GSEA was conducted using the Broad Institute GSEA software [37, 103] (v4.3.2) with weighted enrichment scoring, 1,000 phenotype permutations, and minimum/maximum gene set sizes of 15/500. The resulting term list is in Additional file 4 (Table S3-5). Pathways with FDR *q* < 0.05 were considered significant. To avoid undefined − log₁₀ transformations, FDR *q*-values of zero were replaced with half the smallest nonzero *q*-value, and values were capped at 50 for visualization. Normalized enrichment scores (NES), which represent enrichment scores standardized for gene set size allowing comparisons across gene sets, were used to assess the magnitude and direction of enrichment (positive NES = upregulated, negative NES = downregulated). To identify hub genes from GSEA results, leading edge genes were extracted from all significantly enriched pathways. For each gene, we counted how many pathways included that gene as a leading-edge member (term count). Genes were ranked by term count in decreasing order, with ties broken by the absolute value of DE score. The top 20 genes from each direction were designated as hub genes.

#### Cell-type analysis

To explore cell type–specific transcriptional alterations, cell marker gene sets were obtained from PanglaoDB [49] (https://panglaodb.se; Additional file 5: Table S4-1) and filtered for mouse brain–expressed genes. GSEA was performed using the same parameters as applied for the GO: BP dataset. The resulting term list is in Additional file 5 (Table S4-2).

Bulk RNA-seq deconvolution was performed with MuSiC [50] using the Allen Brain Atlas single-cell RNA-seq dataset as reference (https://portal.brain-map.org/atlases-and-data/rnaseq, accessed on 14th October 2025), which included 31,053 genes across 1,169,320 cells. Hippocampal cells were selected based on the region_label field, resulting in 82,427 cells across 137 types (≥ 5 cells/type; Additional file 5: Table S4-3). A delayed SingleCellExperiment object was generated as the reference, retaining 23,474 genes shared with the bulk RNA-seq dataset. The music_prop function was applied to estimate cell type proportions while accounting for donor-specific variation.

#### Integration and gene prioritization

For pathways significant in both ORA and GSEA, a combined pathway score was computed as:

$$Combined~score~=~\sqrt {{{\left( {ORAscore~} \right)}^2}+~{{\left( {GSEAscore} \right)}^2}} $$

where

$$ORAscore~=~ - lo{g_{10}}\left( {FDR~q{\mathrm{-}}value} \right)~$$

$$GSEAscore~=~\left| {NES} \right| \times ~ - lo{g_{10}}\left( {FDR~q{\mathrm{-}}value} \right)$$

For each gene, a priority score was defined as:

$$\begin{aligned} Priority~score~&=~\left( {pathway~count} \right) \\&\quad \times ~mean\left( {combined~score} \right)\\ &\quad\times ~\left| {DE~score} \right|\end{aligned}$$

Chord plots were generated using the top 20 genes ranked by priority score within each pathway, visualizing the top 5 pathways significant in both ORA and GSEA. All results are collected in Additional file 4 (upregulated pathway list: Table S3-6, downregulated pathway list: Table S3-7, priority scores for upregulated genes: Table S3-8, priority scores for downregulated genes: Table S3-9).

### Cross-species comparison with human schizophrenia

Human postmortem hippocampal RNA-seq data were obtained from the Gene Expression Omnibus (GEO) database (GSE138082) [23]. Metadata and expression matrices were retrieved using the GEOquery [104] (v2.70.0). The dataset comprises 77 samples from 77 unique individuals (39 controls, 38 schizophrenia), each contributing a single hippocampal subfield sample (CA1: *n* = 25; CA3: *n* = 26; DG: *n* = 26). Subjects were non-overlapping across subfields, and the subfield composition was nearly balanced between diagnostic groups (Control: 13/13/13; Schizophrenia: 12/13/13 for CA1/CA3/DG). The cohort included both sexes (Male: 57, Female: 20), with balanced sex composition between diagnostic groups (Male: 30 controls, 27 schizophrenias; Female: 9 controls, 11 schizophrenia). Mouse–human one-to-one orthologs were identified via Ensembl BioMart [105] (biomaRt [106] v2.60.1), yielding 17,176 unique orthologous gene pairs. Differential expression in human samples (control *n* = 39, schizophrenia *n* = 38) was analyzed using edgeR [25] with identical TCC-GUI [95] parameters to the mouse pipeline. Expression filtering, DE scoring and the resampling-based reproducibility test were performed as described above. Supplementary re-analyses incorporating hippocampal subfield (DE score Spearman ρ = 0.970) and sex (ρ = 0.976) as covariates confirmed that the principal findings were robust to these sources of expression variation.

To assess the transcriptomic concordance between species, DE scores of 13,965 orthologous genes retained after filtering were compared with those of mouse genes using Spearman’s ρ and Pearson’s r correlation tests. Overlaps between DEG sets were evaluated using Fisher’s exact test. Pathway enrichment analysis was performed in both species using ORA and GSEA with GO: BP dataset, and conserved pathways were defined as GO terms detected by both approaches in both species. Cross-species pathway similarity was quantified using Spearman’s ρ and Pearson’s r correlations of the combined scores (for full statistical result see Additional file 3, Table S2-3). Protein-protein interaction networks were constructed using STRING (RRID: SCR_005223) [107] website (https://string-db.org/; accessed on 11th February 2026) for genes shared within conserved pathways. Text-mining evidence was excluded, and medium confidence (interaction score ≥ 0.4) was applied. The resulting networks were visualized using Cytoscape software [108] (v 3.10.4). All results are collected in Additional file 6.

## Supplementary Information

Below is the link to the electronic supplementary material.

Supplementary Material 1 (supplemental figures. Figure S1 ~ S4).

Supplementary Material 2 (Table S1-1 Fear conditioning test. Table S1-2 Open field test. Table S1-3 Acule psychostimulant response).

Supplementary Material 3 (Table S2-1 Behavioral test statistics. Table S2-2 DE score validation statistics. Table S2-3 Cross-species statistics).

Supplementary Material 4 (Table S3-1 Df1 dHipp TMM normalized count data. Table S3-2 Df1 dHipp DEG list. Table S3-3 Df1 dHipp ORA GO:BP GOSemSim clustered result: top 300 DEGs. Table S3-4 Df1 dHipp ORA GO:BP GOSemSim clustered result: bottom 300 DEGs. Table S3-5 Df1 dHipp GSEA GO:BP top 300 positively and negatively enriched terms. Table S3-6 Df1 dHipp common pathway identified by both ORA and GSEA: upregulated. Table S3-7 Df1 dHipp common pathway identified by both ORA and GSEA: downregulated. Table S3-8 Df1 dHipp priority score list: upregulated genes. Table S3-9 Df1 dHipp priority score list: downregulated genes).

Supplementary Material 5 (Table S4-1 Curated PanglaoDB dataset. Table S4-2 Df1 dHipp GSEA PanglaoDB top 300 positively and negatively enriched terms. Table S4-3 Allen Brain Atlas single-cell RNA-seq dataset (hippocampal cell)).

Supplementary Material 6 (Table S5-1 GSE138082 human hipp metadata. Table S5-2 GSE138082 human hipp TMM normalized countdata. Table S5-3 GSE138082 human hipp DEG list. Table S5-4 GSE138082 human hipp ORA GO:BP GOSemSim clustered result: top 300 DEGs. Table S5-5 GSE138082 human hipp ORA GO:BP GOSemSim clustered result: bottom 300 DEGs. Table S5-6 GSE138082 human hipp GSEA GO:BP top 300 positively and negatively enriched terms. Table S5-7 Cross species orthologous gene pairs list. Table S5-8 Cross species overlap pathways: upregulated. Table S5-9 Cross species overlap pathways: downregulated).

## Data Availability

The raw and processed RNA-seq data generated in this study have been deposited in the DNA Data Bank of Japan (DDBJ) under accession number PRJDB39695. Human schizophrenia hippocampaldata were obtained from GEO (accession number: GSE138082). All other data supporting the findings of this study are available within the paper and its Additional Files. Source code for DE score calculation and analytical pipelines are available upon request from the corresponding author.

## Abbreviations

22q11.2DS: 22q11.2 deletion syndrome
ANOSIM: Analysis of similarities
ANOVA: Analysis of variance
Apoe: Apolipoprotein E
App: Amyloid beta precursor protein
Aqp4: Aquaporin 4
Bcas1: Breast carcinoma amplified sequence 1
Bmpr2: Bone morphogenetic protein receptor type 2
C3: Complement component 3
Cacna1c: Calcium voltage-gated channel subunit alpha1 C
Cav1: Caveolin 1
Cdkl5: Cyclin-dependent kinase-like 5
Chrna7: Cholinergic receptor nicotinic alpha 7 subunit
Cldn5: Claudin 5
Cntnap2: Contactin-associated protein-like 2
Col9a2: Collagen type IX alpha 2 chain
Comt: Catechol-O-methyltransferase
CS: Conditioned stimulus
DDBJ: DNA Data Bank of Japan
DE: Differential expression
DEG: Differentially expressed gene
dHipp: Dorsal hippocampus
E/I: Excitatory-inhibitory
Ephb2: Ephrin type-B receptor 2
FDR: False discovery rate
Fzd3: Frizzled class receptor 3
GABA: Gamma-aminobutyric acid
Gabra1: Gamma-aminobutyric acid type A receptor subunit alpha1
Gabra3: Gamma-aminobutyric acid type A receptor subunit alpha3
Gabra5: Gamma-aminobutyric acid type A receptor subunit alpha5
Gabrb1: Gamma-aminobutyric acid type A receptor subunit beta1
Gabrb2: Gamma-aminobutyric acid type A receptor subunit beta2
Gabrb3: Gamma-aminobutyric acid type A receptor subunit beta3
Gabrg2: Gamma-aminobutyric acid type A receptor subunit gamma2
Gad2: Glutamate decarboxylase 2
GEO: Gene Expression Omnibus
Gfap: Glial fibrillary acidic protein
GO:BP: Gene Ontology Biological Process
Gpx1: Glutathione peroxidase 1
Gpx4: Glutathione peroxidase 4
Gria1: Glutamate ionotropic receptor AMPA type subunit 1
Grik2: Glutamate ionotropic receptor kainate type subunit 2
Grin1: Glutamate ionotropic receptor NMDA type subunit 1
Grin2a: Glutamate ionotropic receptor NMDA type subunit 2A
Grin2b: Glutamate ionotropic receptor NMDA type subunit 2B
GSEA: Gene set enrichment analysis
Gsk3b: Glycogen synthase kinase 3 beta
IQR: Interquartile range
ITI: Intertrial interval
Kcnj10: Potassium inwardly rectifying channel subfamily J member 10
Mbp: Myelin basic protein
MK-801: (+)-MK-801 hydrogen maleate (dizocilpine)
mPFC: Medial prefrontal cortex
NAc: Nucleus accumbens
NES: Normalized enrichment score
Nes: Nestin
Nlgn1: Neuroligin 1
Nlgn2: Neuroligin 2
NMDA: N-methyl-D-aspartate
Nos1: Nitric oxide synthase 1
Nrxn1: Neurexin1
Ntrk3: Neurotrophic receptor tyrosine kinase 3
ORA: Over-representation analysis
Padi2: Peptidyl arginine deiminase 2
Pafah1b1: Platelet-activating factor acetylhydrolase IB subunit alpha
PCA: Principle component analysis
PERMANOVA: Permutational multivariate analysis of variance
Pmp22: Peripheral myelin protein 22
Ptges: Prostaglandin E synthase
Pten: Phosphatase and tensin homolog
qPCR: Quantitative polymerase chain reaction
Ranbp1: RAN binding protein 1
Reln: Reelin
RNA-seq: RNA sequencing
Rpl: Ribosomal protein large subunit
Rps: Ribosomal protein small subunit
S100b: S100 calcium binding protein B
Scn1a: Sodium voltage-gated channel alpha subunit 1
SEM: Standard error of the mean
Serpinh1: Serpin family H member 1
Tgfbr2: Transforming growth factor beta receptor 2
TMM: Trimmed mean of M-values
Ube3a: Ubiquitin protein ligase E3A
US: Unconditioned stimulus
WT: Wild-type
