# Supplementary material for: Hippocampal transcriptome profiling in a 22q11.2 deletion syndrome mouse model: comparison with human schizophrenia
Source: Mol Brain. 2026 Apr 5;19:41. doi: 10.1186/s13041-026-01300-7 (PMC13188264; doi:10.1186/s13041-026-01300-7)
Supplement: Supplementary file 1 — Supplementary Material 1 (supplemental figures. Figure S1 ~ S4). [file 13041_2026_1300_MOESM1_ESM.pdf]

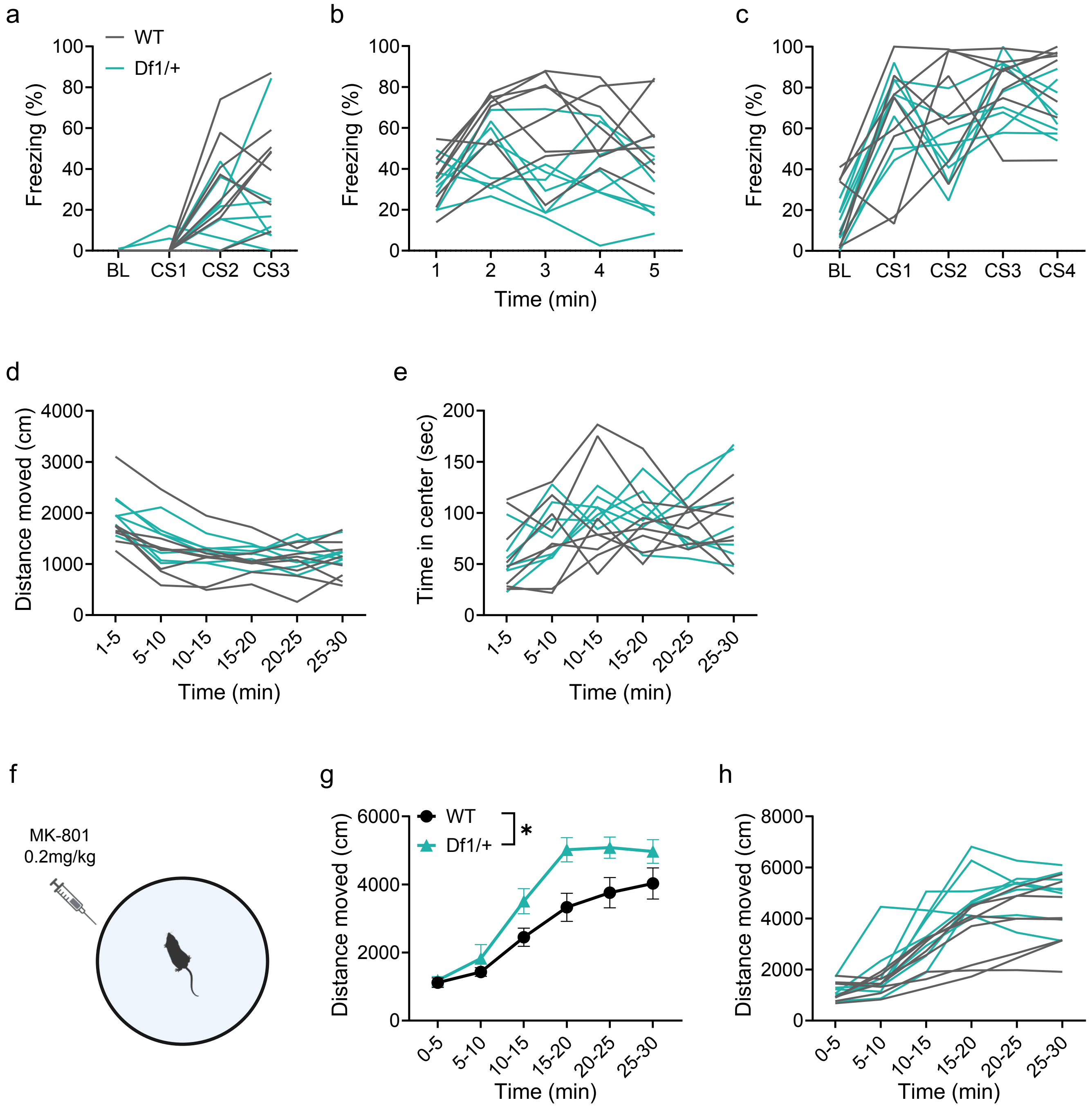

Supplementary Figure 1 Individual behavioral data for fear conditioning, open field, and acute psychostimulant response tests in Df1/+ mice

(a–c) Individual data for the conditioning phase (a), contextual test (b), and cue test (c) of the fear conditioning paradigm. WT n=8, Df1/+ n=8. (d, e) Individual time-series data for distance moved (d) and time spent in the center (e) in the open field test. WT n=8, Df1/+ n=7. (f) Experimental design of the acute psychostimulant response test. Illustration created with BioRender.com. (g, h) Locomotor activity during the acute psychostimulant response test. (g) Mean ± SEM for each genotype; (h) individual trajectories for each mouse. WT n=8, Df1/+ n=7.

a

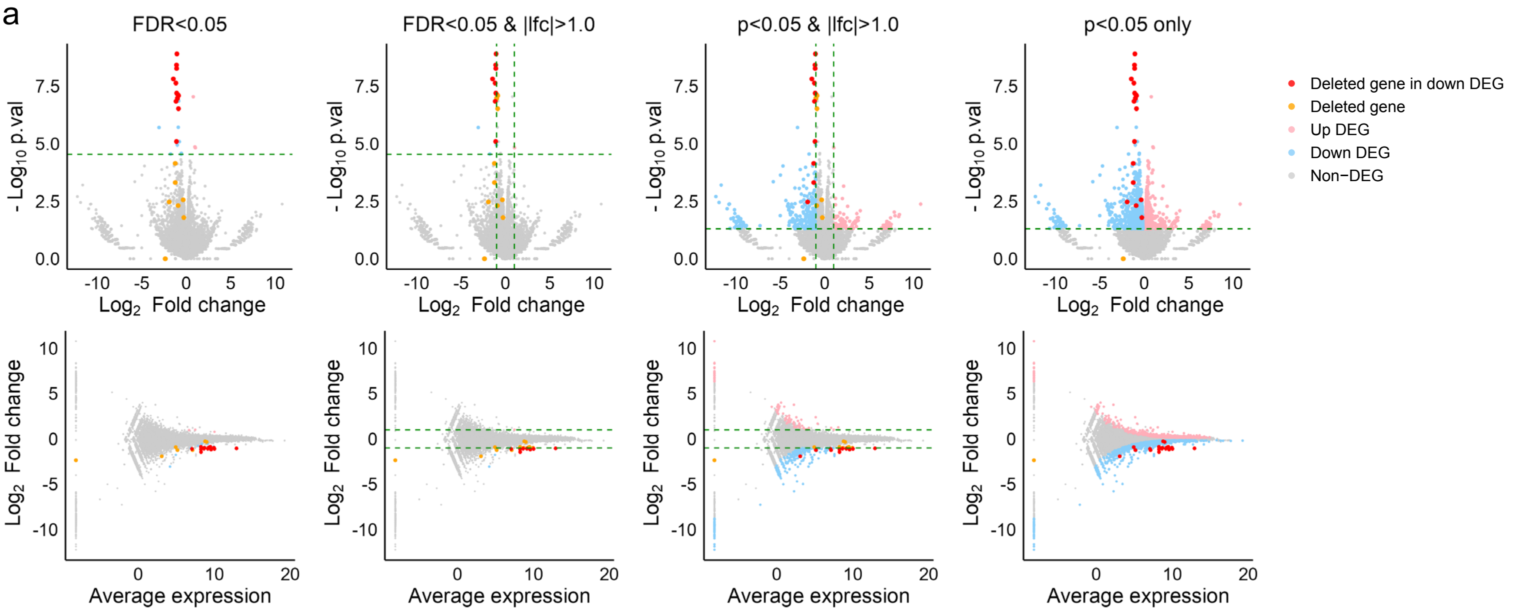

b

Threshold	Deleted genes in DEGs (/22 genes)	No. of DEGs Up	Down	Total	Capture of FDR<0.05 DEGs Up	Down	Overlap with DE score Up	Down
FDR<0.05	13	3	21	24	-	-	3 (100.0%)	21 (100.0%)
FDR<0.05 & |lfc|>1.0	8	1	11	12	1/3 (33%)	11/21 (52%)	1 (100.0%)	11 (100.0%)
p<0.05 & |lfc|>1.0	11	104	277	381	1/3 (33%)	11/21 (52%)	2 (1.9%)	28 (10.1%)
p<0.05 only	19	745	1021	1766	3/3 (100%)	21/21 (100%)	300 (40.3%)	300 (29.4%)
p<0.05 & DE score	16	300	300	600	3/3 (100%)	21/21 (100%)	-	-

c

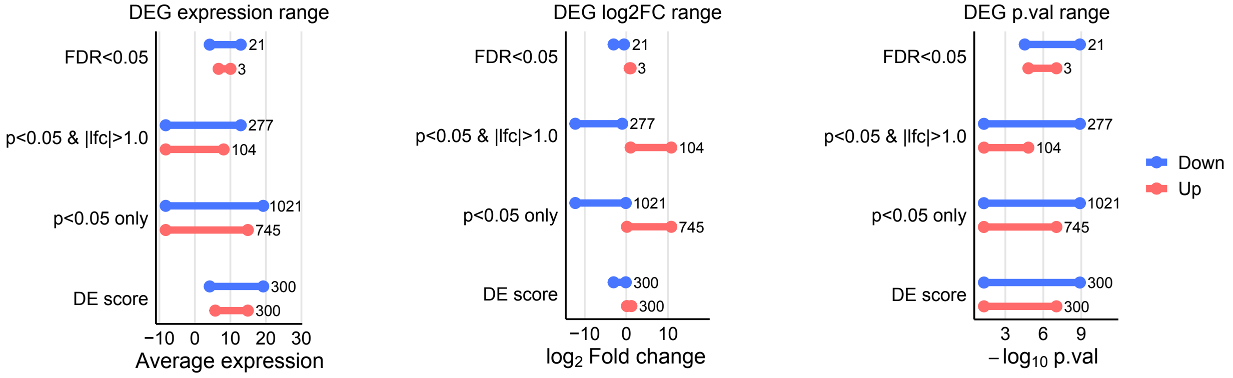

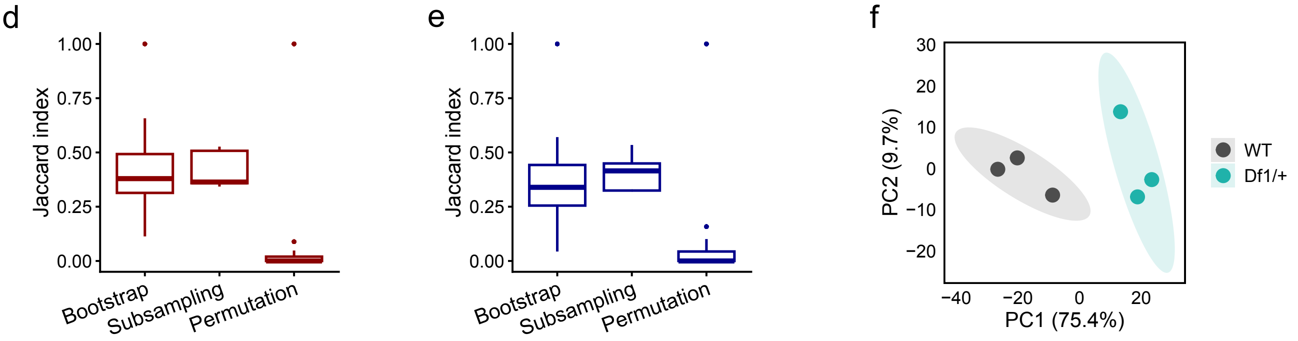

Supplementary Figure 2. Comparison and validation of DEG definition strategies in Df1/+ hippocampal transcriptome analysis

(a) Volcano plots and MA plots for each DEG definition criterion (FDR < 0.05, FDR < 0.05 & $|\log_2 FC| > 1.0$, $p < 0.05$ & $|\log_2 FC| > 1.0$, and $p < 0.05$ only). Red dots denote differentially expressed genes (DEGs) located within the deleted region in Df1/+ mice. Orange dots indicate non-DEGs within the deleted region, pink dots represent the top 300 upregulated DEGs, blue dots represent the bottom 300 downregulated DEGs, and grey dots represent all other genes. (b) Summary table describing the characteristics of each DEG definition strategy. (c) Ranges of average expression level (left), $\log_2$ fold change (center), and $-\log_{10}$ p-value (right) for each DEG definition criterion. Numbers next to each line plot indicate the number of DEGs identified by the corresponding method. (d, e) Reproducibility of DE score–based ranked gene sets evaluated using stratified bootstrap (n = 500; success rate 82.8%), leave-one-out subsampling (n = 100; success rate 100%), and permutation testing (n = 1,000) for the top 300 genes (d) and bottom 300 genes (e). Boxes represent the median and interquartile range (IQR); whiskers extend to 1.5 × IQR, and individual points denote outliers. Higher Jaccard indices indicate greater reproducibility of the gene sets. (f) Principal component analysis (PCA) of the top and bottom 300 genes using TMM-normalized counts. Each point represents a sample. Semi-transparent ellipses indicate the 95% confidence region for each genotype.

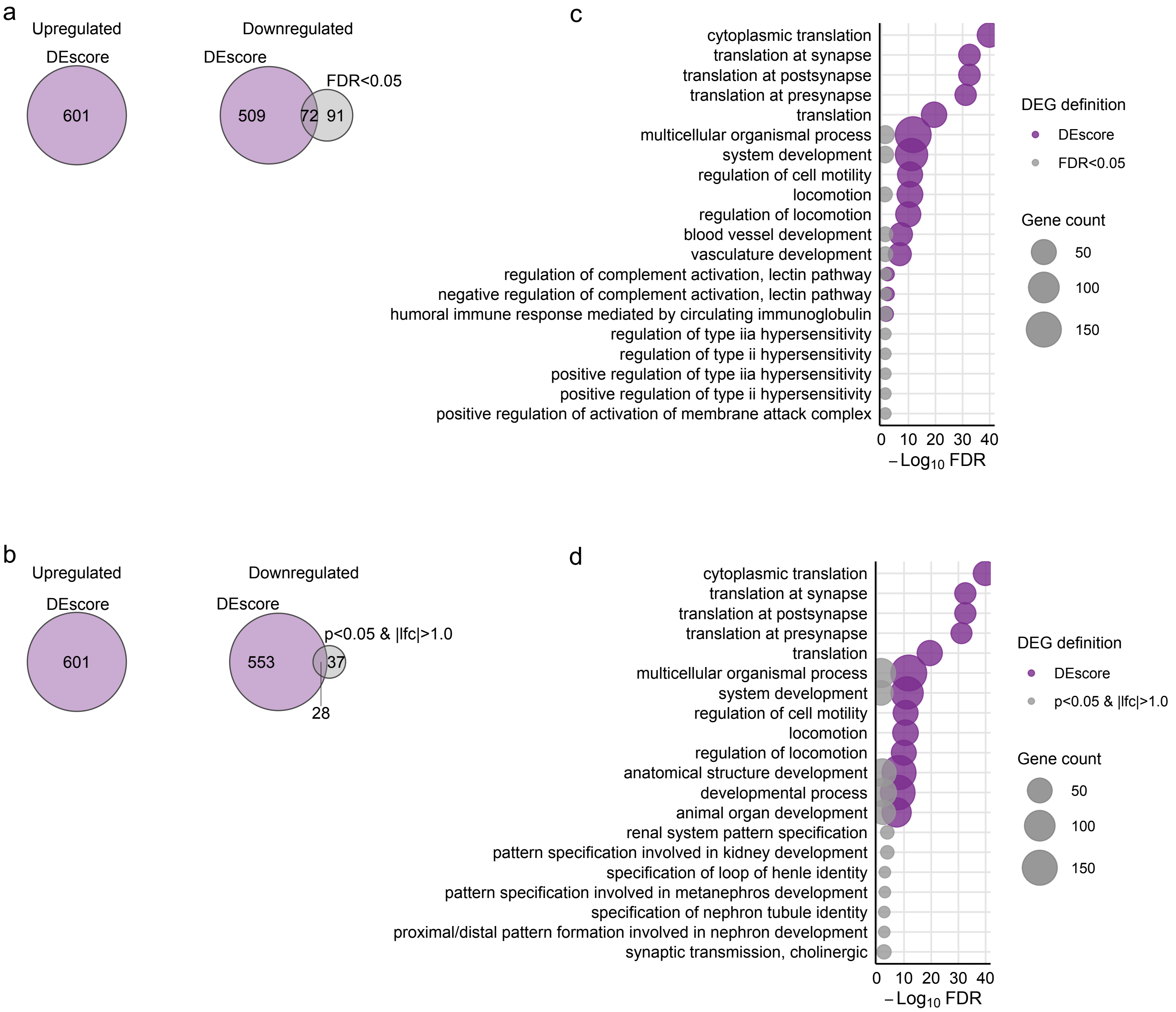

Supplementary Figure 3. Comparison of pathway enrichment results between DE score–based and conventional DEG definitions

(a, b) Venn diagrams illustrating the overlap of enriched terms identified by the DE score–based definition and conventional threshold–based definitions for upregulated (left) and downregulated (right) gene sets. Results are shown for FDR < 0.05 (a) and p < 0.05 & $|\log_2 FC| > 1.0$ (b). (c, d) Dot plots comparing the top 10 downregulated terms identified by the DE score–based definition (purple) and the conventional threshold–based definition (grey). Results are shown for FDR < 0.05 (c) and p < 0.05 with $|\log_2 FC| > 1.0$ (d).

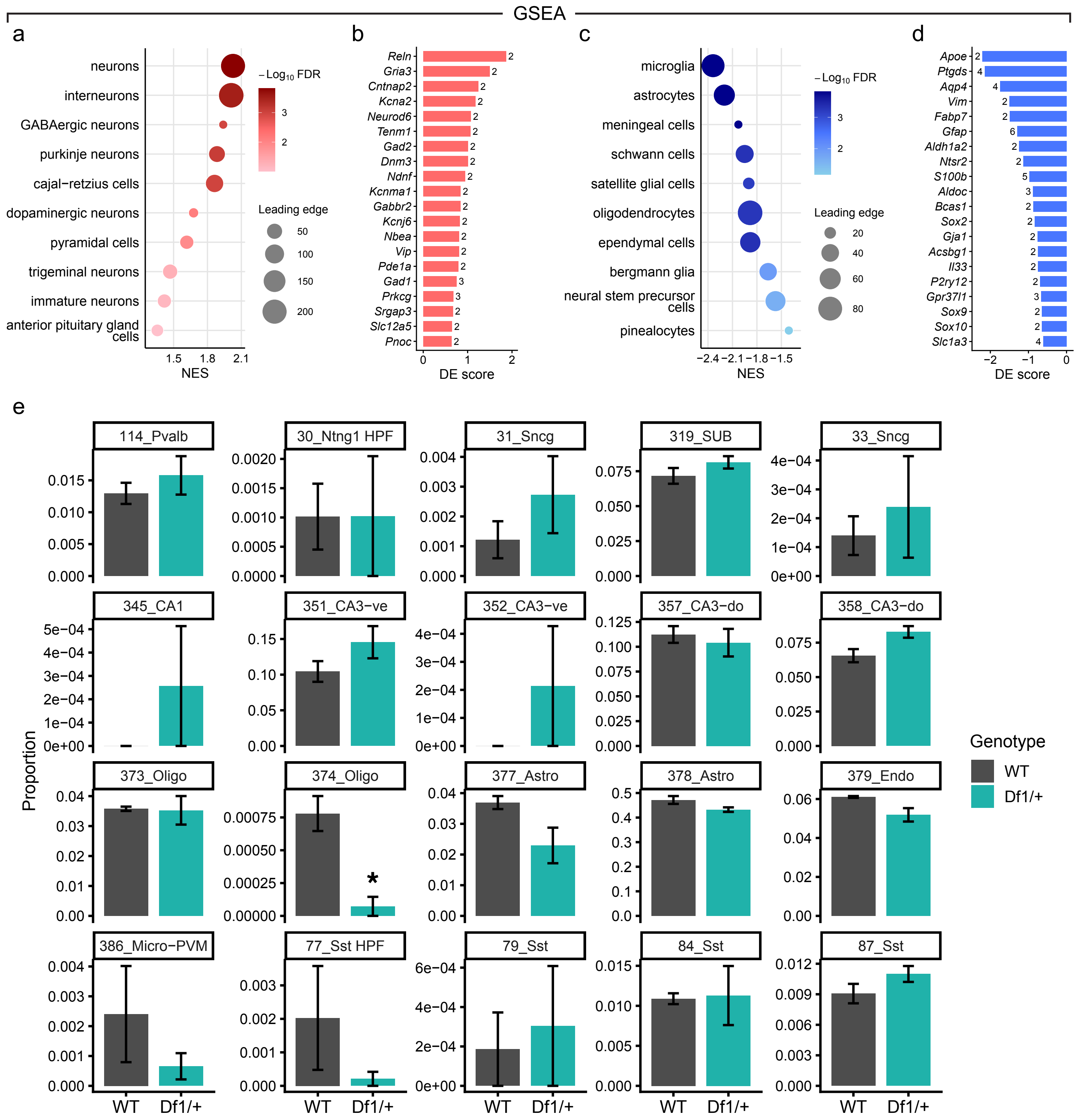

Supplementary Figure 4. Cell-type enrichment and cell composition analysis in the Df1/+ hippocampus

(a, c) Bar plots showing the top 10 positively (a) and negatively enriched (c) cell types identified by gene set enrichment analysis (GSEA) using the curated PanglaoDB dataset. (b, d) Top 20 hub genes for the upregulated (b) and downregulated (d) gene sets. Genes are ranked by term count (the number of pathways in which each gene appears as a leading-edge member), with ties resolved by absolute DE score. Bar length represents the DE score, and the number at the end of each bar indicates the term count. (e) The 20 most abundant cell types based on mean estimated proportions across all samples. Cell-type proportions were estimated using the MuSiC deconvolution method with the Allen Brain Atlas as the reference dataset. Data are presented as mean ± SEM. Statistical comparisons between genotypes were performed for each cell type (Welch's t-test or Wilcoxon rank-sum test depending on normality). *$p < 0.05$.

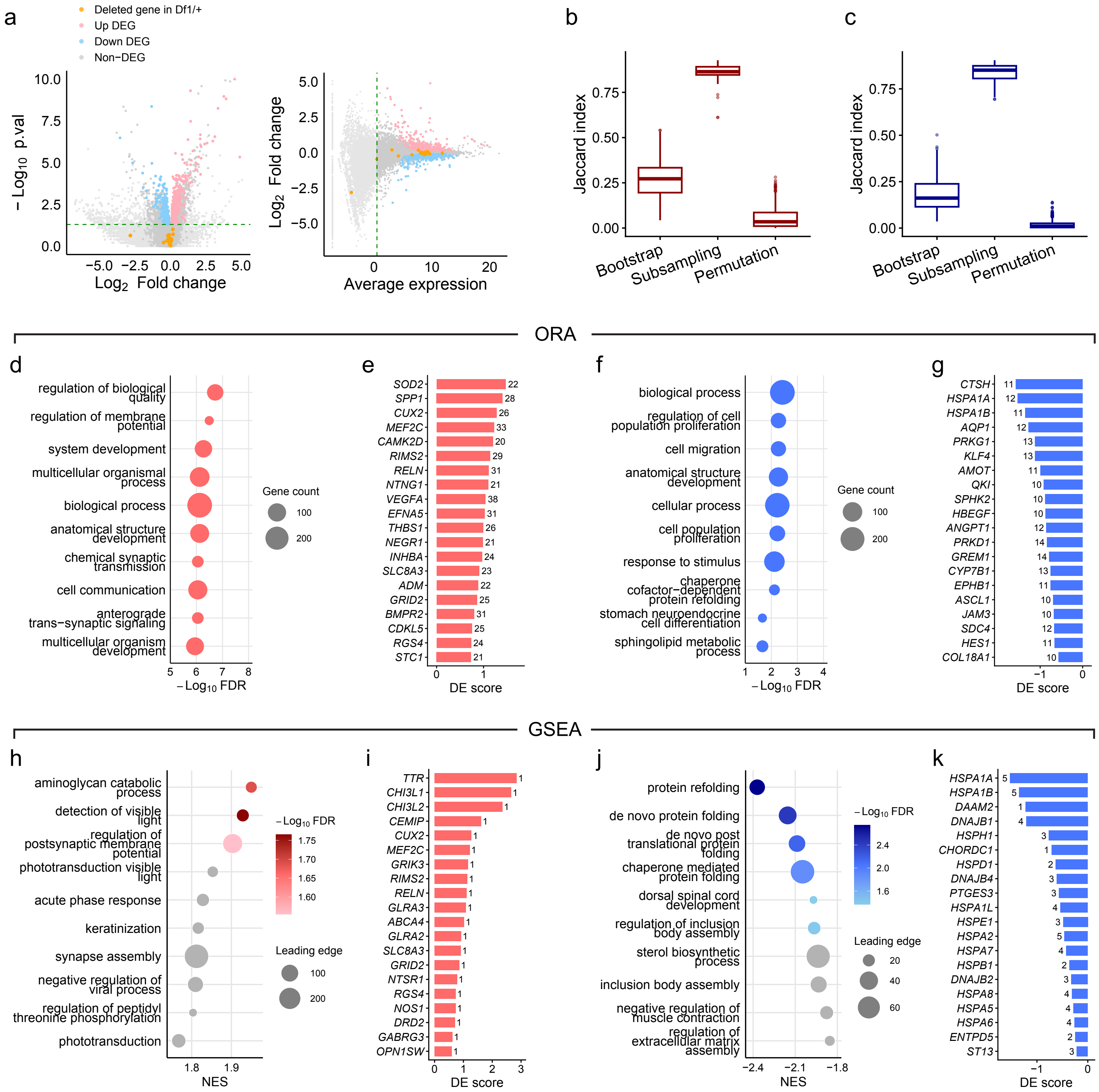

Supplementary Figure 5. Transcriptomic analysis and pathway enrichment in the human schizophrenia hippocampus dataset

(a) Volcano plot and MA plot of filtered genes (29,384 genes; a.value ≥ 0.5) in the human dataset (GSE138082). Orange dots indicate non-DE genes located within the deleted region in Df1/+ mice, pink dots represent the top 300 upregulated DEGs, blue dots indicate the bottom 300 downregulated DEGs, and grey dots represent all other genes.(b, c) Reproducibility of DE score–based ranked gene sets evaluated in the human dataset. Robustness of the top 300 (b) and bottom 300 (c) genes was assessed using stratified bootstrap (n = 500), leave-one-out subsampling (n = 100), and permutation testing (n = 1,000). Boxes represent the median and interquartile range (IQR); whiskers extend to 1.5 × IQR and individual points denote outliers. Higher Jaccard index values indicate greater reproducibility of the ranked gene sets.(d, f) Dot plots showing the top 10 enriched Gene Ontology Biological Process (GO:BP) terms identified by over-representation analysis (ORA) for upregulated (d) and downregulated DEGs (f) in the human dataset. Dot size reflects the number of genes associated with each term.(e, g) ORA-based top 20 hub genes for significantly upregulated (e) and downregulated (g) pathways. Genes are ranked by term count (number of representative terms in which each gene appears), with ties broken by absolute DE score. Bar length represents the DE score, and the number at the end of each bar indicates the term count.(h, j) Dot plots showing the top 10 positively (h) and negatively (j) enriched GO:BP terms identified by gene set enrichment analysis (GSEA). The x-axis represents the normalized enrichment score (NES). Dot size represents the number of leading-edge genes for each term, and dot color indicates statistical significance. (i, k) GSEA-based top 20 hub genes for upregulated (i) and downregulated (k) pathways. Genes are ranked by term count (number of pathways in which each gene appears as a leading-edge member), with ties broken by absolute DE score. Bar length represents the DE score, and the number at the end of each bar indicates the term count.
